# Elastocaloric Waste/Natural Rubber Materials with Various Crosslink Densities

**DOI:** 10.3390/polym15112566

**Published:** 2023-06-02

**Authors:** Nicolas Candau, Adele Zimny, Eduard Vives, Maria Lluïsa Maspoch

**Affiliations:** 1 Departament de Ciència i Enginyeria de Materials (CEM), Escola d’Enginyeria Barcelona-Est (EEBE), Universitat Politècnica de Catalunya BarcelonaTech (UPC), Av. Eduard Maristany 16, 08019 Barcelona, Spain; adele.zimny9@etu.univ-lorraine.fr (A.Z.); maria.lluisa.maspoch@upc.edu (M.L.M.); 2 Departament de Física de la Matèria Condensada, Facultat de Física, Universitat de Barcelona, Martí i Franquès 1-11, 08028 Barcelona, Spain; eduard@fmc.ub.edu; 3 Institute of Complex Systems (UBICS), University of Barcelona, Martí i Franquès 1-11, 08028 Barcelona, Spain

**Keywords:** waste rubber, natural rubber, elastocaloric effect, heating/cooling devices

## Abstract

The characterization of the mechanical behavior of elastocaloric materials is essential to identify their viability in heating/cooling devices. Natural rubber (NR) is a promising elastocaloric (eC) polymer as it requires low external stress to induce a wide temperature span, ΔT. Nonetheless, solutions are needed to further improve DT, especially when targeting cooling applications. To this aim, we designed NR-based materials and optimized the specimen thickness, the density of their chemical crosslinks, and the quantity of ground tire rubber (GTR) used as reinforcing fillers. The eC properties under a single and cyclic loading conditions of the resulting vulcanized rubber composites were investigated via the measure of the heat exchange at the specimen surface using infrared thermography. The highest eC performance was found with the specimen geometry with the lowest thickness (0.6 mm) and a GTR content of 30 wt.%. The maximum temperature span under single interrupted cycle and multiple continuous cycles were equal to 12 °C and 4 °C, respectively. These results were assumed to be related to more homogeneous curing in these materials and to a higher crosslink density and GTR content which both act as nucleating elements for the strain-induced crystallization at the origin of the eC effect. This investigation would be of interest for the design of eC rubber-based composites in eco-friendly heating/cooling devices.

## 1. Introduction

Harmful vapor compression devices using hydrofluorocarbons (HFCs) with huge Global Warming Potential (GWP) dominate the cooling technology in many sectors (e.g., domestic, industrial, and transportation cooling devices). Alternative heat exchange processes with lower GWP, using eco-friendly and highly caloric materials, need to be developed. Nonetheless, there are still a few heating/cooling systems based on elastocaloric (eC) of bio-based materials such as natural rubber (NR).

Natural rubber is a type of elastomer usually derived from the sap or latex of the Hevea brasiliensis tree, while alternative sources, such as dandelion or guayule are promising substitutes [1]. NR is composed of polyisoprene molecules and contains a naturally occurring network due to the presence of phospholipids and proteins [2]. After its coagulation and drying, it is milled and cured by using various types of vulcanizing agents, such as peroxide or sulfur [3].

Natural rubber exhibits unique properties such as high elasticity, excellent resilience, and good tear resistance, making it ideal for various applications [4]. Due to its high tensile strength [5], and excellent fatigue behavior [6], it is widely used in the automotive industry for tires and rubber components, in manufacturing rubber gloves, adhesives, and footwear [7]. Its elastocaloric performance was not used yet, so it can be used in an industrial application. Nonetheless, the cooling performance of NR was described in prototypes using a rubber pipe [8] or thin membranes [9]. Caloric effects in NR or NR-based composites were found to be mostly originated from their ability to crystallize under strain (SIC). However, several drawbacks explain the lack of popularization of such prototypes. For instance, the large deformation needed to initiate heating/cooling in the rubber prevents this material from being adequately used in compact devices [10]. Nonetheless, a pre-stretching of the NR specimen and a fine selection of the cyclic strain range where SIC and melting occurs during loading and unloading, respectively, may be used as a solution to drastically reduce the deformation amplitude of the cycles in the heating/cooling machine. It was shown that applying or releasing up to 3 MPa of mechanical stress can produce heating/cooling of NR samples with a maximum temperature span of almost 20 °C [11].

SIC is known to improve the fatigue life of NR and NR-based materials [12]. SIC is, hence, necessary to work under cyclic conditions for the adequate functioning of eC prototypes [13]. A series of 10^5^–10^6^ cycles under a large strain amplitude (500%) and even up to 10^7^ cycles upon a moderate strain amplitude (200%) can be applied [14]. The effect of temperature on fatigue life has also been investigated. It was found that the largest lifetime was obtained for NR samples tested at 23 °C. As the temperature increased, SIC ability decreased and the lifetime decreased until it totally disappears at 110 °C [15,16]. Despite large number of studies about the effect of the loading and temperature conditions, there is still a need to understand and optimize the eC effect occurring under cyclic conditions in NR-based composites using bio-based or recycled fillers.

SIC and mechanical reinforcement of natural rubber-based composites has been extensively studied [17,18,19,20,21]. The incorporation of rigid filler particles, such as carbon black or silica, in the rubber matrix expectedly results in the increase in the elastic modulus. The filler–rubber interactions result in additional crosslinks that further reinforce the resulting composite. Finally, the large strain behavior is governed by the limited chain extensibility, which is accelerated in the presence of rigid fillers due to a strain-amplification effect [22].

Particle size, structure, and, essentially, surface characteristics are filler morphology parameters that are expected to largely influence the performance of natural rubber materials. For instance, coupling agents in silica-filled rubber can create connections between the polymer matrix and the surface of fillers, leading to improved reinforcement. Owing to their intrinsic properties, and their interaction with the rubber matrices, carbon black is useful to improve the durability, strength, and wear resistance of tires, while silica is used for green tires or electrical insulation.

Reinforcing fillers commonly incorporated into NR, such as carbon black fillers [23], silica [24], graphene oxides [25], or clay [26], were also found to act as nucleating agents for SIC in NR. The incorporation of renewable and/or recycled fillers such as non-crystallizable waste rubber [27,28], into NR generally shows a deterioration of the tensile strength. Contrarily, the addition of crystallizable wastes, such as waste chloroprene rubber (CR) [29], waste latex [30], or ground tire rubber (GTR) [31], was found to enhance the mechanical reinforcement of the resulting composites. The use of 20 wt.% of GTR in an NR matrix shows optimum eC properties [32,33]. Such reinforcement was found to originate from the nucleating effect of waste fillers on SIC in NR [34]. Nonetheless, the literature does not show any intent of improvement of the resulting eC effect in the NR/GTR composites that could arise from an optimization of the specimen dimensions or vulcanizing agent that both would influence the crosslink density of the corresponding composite, which would directly affect the eC effect. Moreover, the improvement of NR-based composites processing and testing methods to improve the materials’ longevity and the eC effect on these materials in real conditions of solicitation that can be found in potential eC prototypes still need to be investigated.

In this paper, we prepared vulcanized natural/waste rubber blends as efficient eC materials. The eC effect in NR/GTR blends with various crosslink density has been studied. This latter parameter was chosen to be studied by varying the specimen geometries (thickness), the crosslinking agent (DCP), and the GTR content. DCP is chosen owing to its high-temperature resistance, which is required when targeting the application where a large number of mechanical cycles involving temperature changes is required (due to elastocaloric effect and/or damage). Several testing conditions were tested, from single loading to interrupted cyclic loading and finally, continuous successive cycles that would fit with conditions of solicitation in possible eC prototypes.

## 2. Materials and Experiments

### 2.1. Materials and Processing

The natural rubber (NR) of this study is an SMR (Standard Malaysian Rubber) CV60 (Mooney Viscosity ML 1 + 4, 100 °C: 55–60), supplied by the company Akrochem (USA), with 0.15% of hydroxylamine added to the latex stage to prevent the raw rubber stiffening while storing. Ground tire rubber (GTR) was supplied by the company J. Allcock and Sons Ltd. (Manchester, UK) using the transformation of used tire buffing into finer rubber crumbs via a controlled cryo-grinding. GTR is composed of rubber (85 wt.% of natural rubber and 15 wt.% of Styrene Butadiene Rubber) and carbon black, CB. It also contains additives from sulfur vulcanization (see SEM-EDX images in ref. [35]). The GTR crumbs are free of contaminants such as textile, metal, and road dirt. The GTR particles were subsequently sieved using a vibratory sieve shaker Analysette 3 (Idar-Oberstein, Germany) with a mesh size of 230 s (size < 63 μm). The NR was masticated inside the chamber of an internal mixer Brabender Plastic-Corder W50EHT (Brabender GmbH and Co., Duisburg, Germany), volume chamber 55 cm^3^ at a temperature of 80 °C for 5 min and a rotation speed of 40 rpm. After 5 min of mastication, the GTR was added. After 5 more minutes, the vulcanizing agent dicumyl peroxide (DCP) was added (1.5 or 2 wt.% of the NR) and mixed for 5 min. The masterbatch containing NR, GTR, and DCP was vulcanized under a hot press according to the estimated optimal time at 170 °C under 4 MPa. Table 1 gives the composition and properties of the prepared materials. It has been shown [36] that the introduction of carbon black fillers at a content of 33 wt.% (50 phr) did not significantly modify the curing time in the NR matrix. In our case, the quantity of carbon black (around 45 wt.% of the GTR) is 9 and 13.5 wt.% of the overall weight of the blend, respectively. In our series of blends, the curing time has been determined from the literature, where DCP-cured natural rubber had been vulcanized at various temperatures and DCP content ranging from 0.5 to 3 phr [37,38,39]. The plates extracted from hot press have different thickness, as indicated in Table 1. From each plate, several tensile specimens were extracted. The plates have an average value of thickness, and the error bar has been determined from the measure of the thickness of the tensile specimen (at least 7 for each plate). 

### 2.2. Swelling

The rubber was immersed in cyclohexane for 72 h. After 72 h, the swollen mass was measured. The rubber was then placed under a hood at room temperature for 72 h to remove the solvent. The mass of the dry samples was then measured. The average network chain density has been calculated from swelling experiments and the Flory–Rehner equation [40]:

(1) $$\upsilon =\frac{ln\left(1-v_{2}\right)+v_{2}+\chi_{1}v_{2}{}^{2}}{V_{1}\left(-v_{2}{}^{\frac{1}{3}}+\frac{2}{f}v_{2}\right)}$$

where *V*_1_ = 108 cm^3^/mol^−1^ is the molar volume of the solvent (cyclohexane), *χ*_1_ is the Flory–Huggins polymer solvent dimensionless interaction term (*χ*_1_ = 0.363 in the case of NR-cyclohexane [41]). $v_{2}=1/Q_{r}$, with *Q_r_* the swelling ratio of the rubber matrix. *Q_r_* = *V/V*_0_ where *V* and *V*_0_ are the volumes of the rubber network at swelling equilibrium and after swelling and drying, respectively. The ratio 2/*f* is associated with the phantom model that assumes spatial fluctuation of crosslinks (non-affine) used for high deformation ratios. *f*, the crosslink functionality, is chosen equal to 4. For filled compounds, the Kraus correction [42] is used to account for the contribution of filler in the swelling ratio:

(2) $$Q_{r}=\frac{Q_{c}-\varphi }{1-\varphi }$$

with *φ* is the volume fraction of fillers and *Q_c_* is the swelling ratio of the composite. Equation (2) assumes non-adhesion of the fillers to the rubbery matrix, hence, creating a vacuole. In our case, the filler volume fraction has been calculated as the quantity of carbon black particles contained in the NR/GTR that was calculated from thermogravimetry analysis (TGA) in ref. [35]. Three tests were performed for each material.

The soluble fraction, defined as the relative loss of weight of the dry material before and after the swelling test, has been found to equal 5% (+/−0.2%), 4.4% (+/−0.02%), and 4.2% (+/−0.02%) for NR, NR/GTR20, and NR/GTR30. It slightly decreases with the quantity of waste. Moreover, no waste has been detected in the solvent after the completion of the swelling test, suggesting a good adhesion between the polymer matrix and the waste, under swelling conditions.

### 2.3. Slow Strain Rate Uniaxial Tensile Stretching (UTS)

Dogbone-shaped specimens were extracted from hot molded sheets by die-cutting with a specimen preparation punching machine (CEAST). The specimen dimensions are the following: A thickness varying from 0.62 to 2.57 mm (see Table 1), a constant width of 4 mm, and a constant length of 15 mm. Uniaxial tests were performed on a universal testing machine Zwick/Roell equipped with a 5 kN force sensor at room temperature and a constant crosshead speed of 100 mm/min. The correct specimen deformation was estimated by measuring the local deformation between two white lines drawn in the central part of the specimen, separated by an initial distance of 10 mm and orthogonal to the specimen tensile axis. Five tests were performed for each material.

### 2.4. High Strain Rate In-Situ Infrared Thermography (In-Situ IR)

Room temperature uniaxial tensile tests were performed on a universal testing machine Zwick/Roell (Z005) equipped with a 5 kN force sensor. Two types of tests were performed: A single loading up to failure and a cyclic test. The single loading was performed with a crosshead speed of 3000 mm/min—corresponding to a nominal strain rate of 3.3 s^−1^, according to the specimen’s initial length. The cyclic test comprises 4 phases: (1) A single loading with a crosshead speed of 3000 mm/min—up to a deformation of 480%, (2) a relaxation step in the deformed state for one minute, (3) unloading with a crosshead speed of 3000 mm/min down to zero force, and (4) a relaxation for one minute. The temperature field on the front face of the samples was recorded using an Infra-red (IR) camera (InfraTech ImageIR^®^ 8800) equipped with a Mercury–Cadmium–Telluride (MCT) detector with a temperature resolution at 30 °C higher than 0.035 K. By comparing measurements in samples covered with high emissivity paint and unpainted samples, we have concluded that there are no deviations in the temperature measurements due to low surface emissivity. Moreover, special care was also taken to prevent IR reflections by covering the whole experiment with a homogeneous polystyrene shield. The distance between the IR camera and the specimen was equal to 65 mm resulting in a size of the observation zone of 160 × 500 pixels^2^ (32 × 100 mm^2^) and a pixel size of 200 μm. Image data was synchronized with analogical data of the tensile test machine (time, force, and displacement between the grips). The acquisition frequency of the IR images of 100 Hz captures temperature fields for each strain increment of 8% during loading/unloading or equivalently for each time increment of 10 ms. The IRBIS 3.1 software (InfraTech ImageIR^®^ 8800) was used to extract punctual temperature values along the specimen’s longitudinal axis. The central part of the specimen where the temperature data was extracted shows a rather homogeneous temperature field at the mm scale. Regarding the complexity of the in-situ infrared experiments and their analysis, only one test was performed for each material.

## 3. Results and Discussion

### 3.1. Tensile Behavior during Slow Strain Rate Single and Cyclic Loading

**Figure 1:**
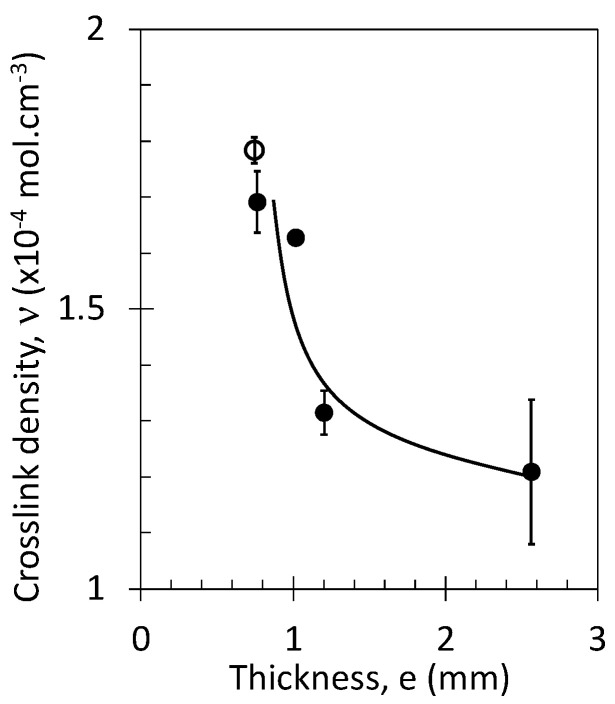
Crosslink density as a function of the specimen thickness for specimen hot pressed for 5 min and taken off the plate to be cooled at ambient temperature (unfilled symbol), for specimen hot pressed for 5 min and subsequently cooled down to ambient temperature at a cooling rate of −50 °C/min.

The mechanical properties of the vulcanized NR and NR/GTR blends, such as the elastic modulus, large strain hardening, and strain-induced crystallization, are partly ruled by the crosslink density of the rubber chains (see Section 2) created during vulcanization. The crosslink density is shown for NR samples of various thicknesses (Figure 1). The thinnest plates appear to be the most vulcanized. The lower crosslink density in the thickest specimens may be due to an incomplete vulcanization process, possibly induced by a heterogeneous heating profile along the material’s thickness axis during the curing under hot press. Two materials with similar thickness (0.75 mm) were vulcanized following two different procedures: (1) The NR was directly taken off the press and put immediately at ambient temperature, and (2) the NR was cooled in the press down to ambient temperature at a cooling rate of −50 °C/min. The longest exposure to high-temperature results in a slight increase in the average crosslink density (Figure 1). The vulcanization time and the specimen geometries (thickness), via a modification of the crosslink density of the NR and NR/GTR blends, are expected to impact the tensile, crystallization, and elastocaloric properties, as will be discussed in the following section. 

**Figure 2:**
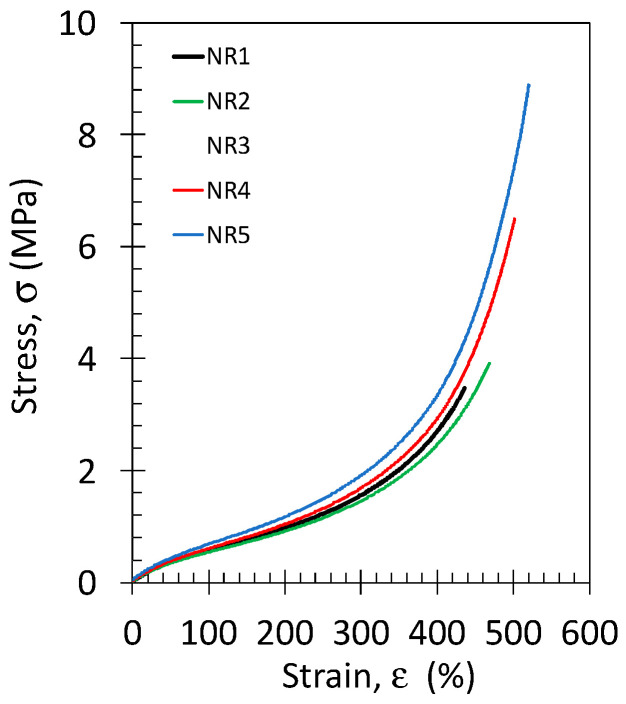
Stress–strain curves preformed at room temperature and at the strain rate of 0.11 s^−1^ for the NR specimen of different thicknesses, as indicated in Table 1.

Tensile properties at room temperature (21 °C) and slow strain rate (0.11 s^−1^) of the NR and NR/GTR blends with various crosslink densities are presented (Figure 2). The curves show a hyperelastic behavior, with a linear elastic regime followed by stress softening due to viscoelasticity and a large strain reinforcement before failure. The large strain reinforcement may be attributed to different mechanisms: Reaching the limit extensibility of the rubber chains and/or the occurrence of strain-induced crystallization where highly oriented crystals play the role of reinforcing fillers. The curves show an increasing reinforcing effect while decreasing the sample thickness, which may be related to differences in the crosslink density, as commented above (Figure 1). Higher crosslink density may indeed facilitate the large strain reinforcement due to faster reaching of the limit chain extensibility, as described by Arruda and Boyce [43]. Moreover, the strain-induced crystallization, which was demonstrated to be favored (higher-level crystallinity versus strain) while increasing crosslink density in peroxide-cured NR [44].

The elastic modulus in the NR and NR/GTR blends can be approximated by modeling the stress–strain relation of the molecular chains following the Gaussian approximation [45], $\sigma =E/3\times (\lambda -1/\lambda^{2}$) where σ and *E* are the tensile stress and elastic modulus, respectively, and the stretching ratio λ is equal to 1 + ε, with ε the deformation (or strain). By using the least square method at a limited range of deformation (*ε* = 200%) between the experimental curve and the model curves, the best-fitting Gaussian curve enables the calculation of the elastic modulus. 

**Figure 3:**
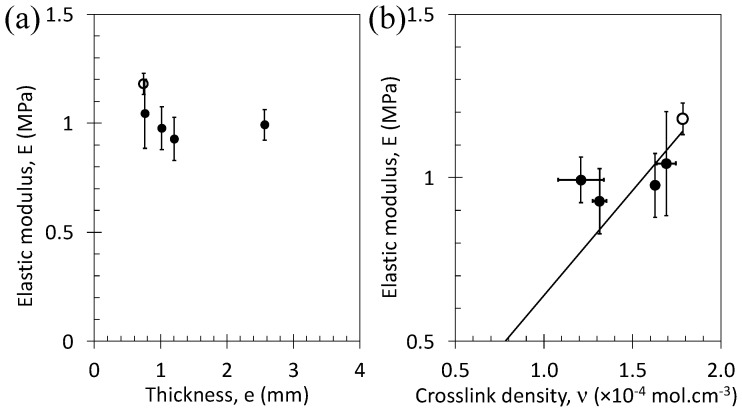
Elastic modulus from tensile tests at room temperature and a strain rate of 0.1 s^−1^ as a function of the thickness of the rubber specimen (**a**) and as a function of the crosslink density estimated from swelling experiments (**b**). Filled symbols correspond to specimens hot pressed for 5 min and taken off the plate to be cooled at ambient temperature, and unfilled symbols correspond to a specimen hot pressed for 5 min and subsequently cooled down to ambient temperature at a cooling rate of −50 °C/min.

The reduction of specimen thickness from 1.2 to 0.7 mm is clearly associated with an increase in the elastic modulus (Figure 3a). Moreover, the elastic modulus is increased with increasing curing time. Within the Gaussian approximation, the elastic modulus is written as *E*/3 = *nRT* with R the gas constant (J·mol^−1^ K^−1^), T the temperature (K), and *n* the crosslink density (mol·cm^−3^). *n* estimated from the tensile tests, using the previous relation, is not necessarily identical to *n* estimated from swelling, possibly due to the presence of topological constraints and/or entanglements that act differently in the function of the techniques used [46]. However, a linear trend is usually found between both *n* values [47]. This trend is confirmed for our series of materials except for the thickest sample that deviates from the linear trend (Figure 3b). 

**Figure 4:**
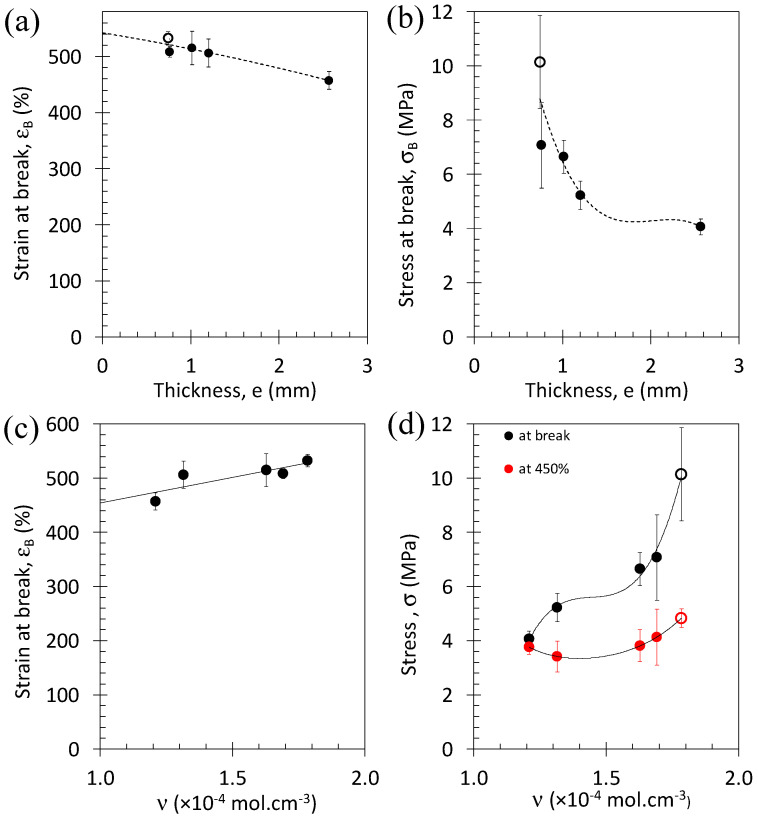
Strain at break versus materials thickness (**a**), stress at break versus materials thickness (**b**), strain at break versus materials crosslink density (**c**), and stress at break and stress at 450% versus crosslink density (**d**). Filled symbols correspond to specimens hot pressed for 5 min and taken off the plate to be cooled at ambient temperature, and unfilled symbols correspond to a specimen hot pressed for 5 min and subsequently cooled down to ambient temperature at a cooling rate of −50 °C/min.

The effect of the specimen thickness and curing time on the large strain properties are investigated by reporting the strain at break, the stress at break, the stress at *ε* = 450% (Figure 4a–d). The decrease in the specimen thickness and an increase in the curing time are both associated with an increase in both strain at break (Figure 4a) and stress at break (Figure 4b). Conversely, from the relationship between specimen thickness and crosslink density (Figure 2), the stress and strain at break are increased with the crosslink density (Figure 4c,d). 

Expectedly, the stress at break increases with the crosslink density at small amounts of crosslinks but decreases for over-crosslinked rubbers [48]. As a result, the stress at break is usually optimum at some intermediate crosslink density. In our series of materials, the higher the crosslink density, the higher the stress at break, suggesting that our series of materials have crosslink densities below the optimum. More surprisingly, the positive effect of the crosslink density on strain at break is contrary to expectations for the literature [48]. Expectedly, the higher crosslink density, the smallest distance between crosslinks, the more rapid chain extension and, hence, a more rapid breakage of the material should have been observed. This discrepancy is likely explained by an incomplete vulcanization process for the specimens with the highest thickness, which might have induced heterogeneities in the curing process (in the thickness profile), resulting in a deteriorated tensile property. On the contrary, specimens showing largest curing time and smaller thickness are expected to be more homogeneously cured. 

Novel series of rubber blends were prepared by using the lowest specimen thickness and a larger amount of crosslinking agent, as they are both suspected to improve the crystallization ability of the natural rubber to crystallize under strain, thanks to respectively (i) an increase in the average crosslink density where crosslinks act as nucleation points, and (ii) the higher homogeneity of the vulcanization allowing strain localization and premature materials failure. In addition, to respond to the increasing demand for high added value materials based on materials recycling, the class of tested materials has been extended to natural rubber containing waste rubber from the pneumatic industry, co-called ground tire rubber (GTR). Finally, the effect of the quantity of crosslinking agent has been investigated to further optimize the tensile behavior and possibly the elastocaloric (eC) effect. 

**Figure 5:**
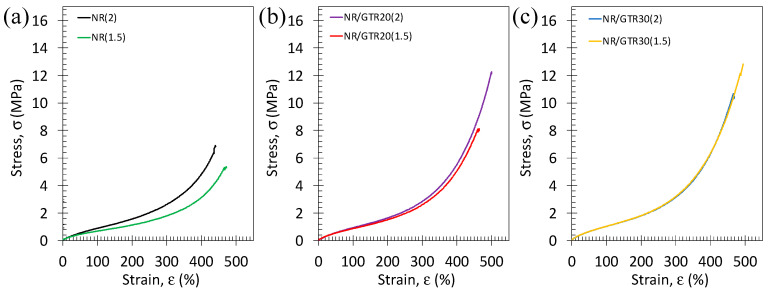
Stress–strain curves preformed at room temperature and at the strain rate of 0.11 s^−1^ for the NR (**a**), NR/GTR20 (**b**), and NR/GTR30 (**c**) for two contents of the crosslinking agent, dicumyl peroxide (DCP): 1.5 wt.% and 2 wt.% of NR.

Tensile curves at room temperature and slow strain rate (0.11 s^−1^) of NR and NR/GTR composites with two different amounts of crosslinking agents are shown (Figure 5). They expectedly show a hyperelastic behavior, typical for vulcanized rubber materials. In NR, the higher the content of crosslinking agent, the more pronounced the large strain reinforcement. This effect, however, tends to decrease while adding GTR particles and even vanishes for a quantity of GTR of 30 wt.% of GTR. 

**Figure 6:**
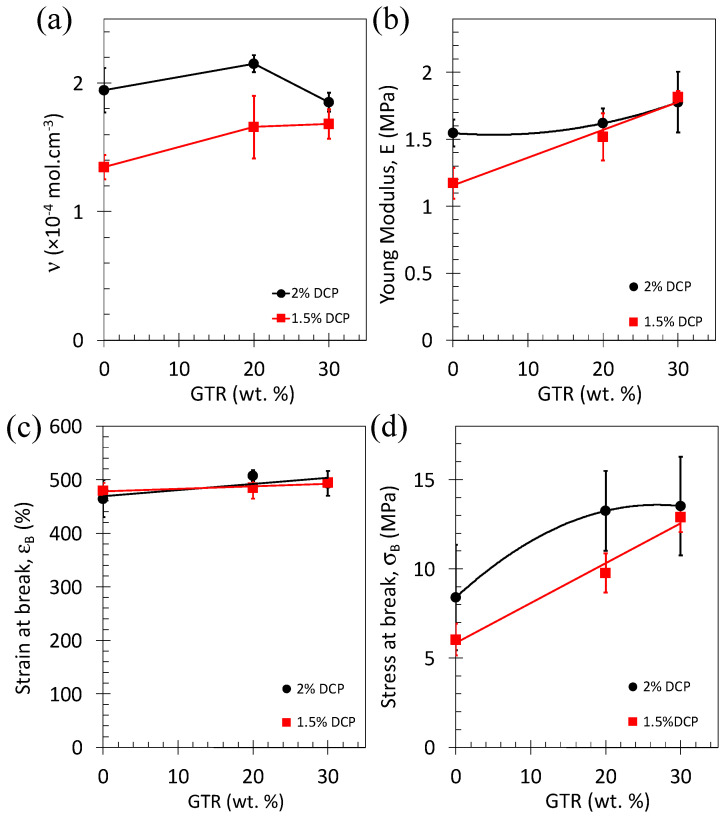
(**a**) Crosslink density, (**b**) elastic modulus, (**c**) strain at break, and (**d**) stress at break as a function of the content of ground tire rubber (GTR) for two series of NR/GTR blends containing 1.5 and 2 wt.% DCP.

Such behavior is reflected in the crosslink density, *n*, and mechanical parameters as shown in Figure 6. *n*, E, and stress at break are increased while increasing the DCP content from 1.5 to 2 wt.% of the NR (Figure 6a,b,d). As the strain at break is not affected by the DCP content (Figure 6c), the increased stress at break is due to higher mechanical reinforcement expectedly due to the formation of a higher number of crosslinks at higher DCP content. While adding GTR particles, the effect of the DCP on the crosslink density and tensile properties vanishes, consistent with the observed similitude of tensile curves for the NR/GTR30 blends (see Figure 5c). This behavior may have several origins: (i) A weak interfacial strength between the NR matrix and the GTR particles due to incomplete vulcanization at the NR/GTR interface or (ii) an underestimate of the amount of DCP required to vulcanize the NR/GTR blends. One may note that the DCP amount was calculated based on the assumption that only the NR matrix and the GTR surface were reactive to DCP (the internal volume of GTR was assumed to be non-reactive). If this assumption is incorrect, this means that the highest the GTR content, the lowest is the DCP amount available to vulcanize both the NR and GTR, hence resulting in an underestimate of the required DCP amount. 

Vulcanized natural rubber-based blends were found to show improved mechanical reinforcement by (i) reducing the specimen thickness, (ii) increasing the content of the curing agent, (iii) increasing the quantity of waste particles. All three parameters improve the average crosslink density as well as its homogeneity. An important role of the crosslinks is to increase the ability of the rubber phase to crystallize under strain through their presence as pivot points for the strain-induced crystal nucleation at the origin of the reinforcing effect. In the following, we aim to make use of this crystallization behavior in terms of heating/cooling response. This has been done by applying high strain rate tensile tests (near adiabatic conditions) where crystallization and melting are accompanied by heat exchanges.

### 3.2. Elastocaloric Behavior during High Strain Rate Single and Cyclic Loading 

The NR/GTR blends are tested during high strain rate (3.33 s^−1^) single loading or cyclic loading. Cycles were applied to detect the heating induced by the loading phase caused by strain-induced crystallization but also to detect the cooling induced by the unloading phase of the cycle caused by the melting of strain-induced crystals. Relaxation phases were applied right after loading and unloading to allow return to the equilibrium temperature and, hence, better differentiate both heating and cooling effects.

**Figure 7:**
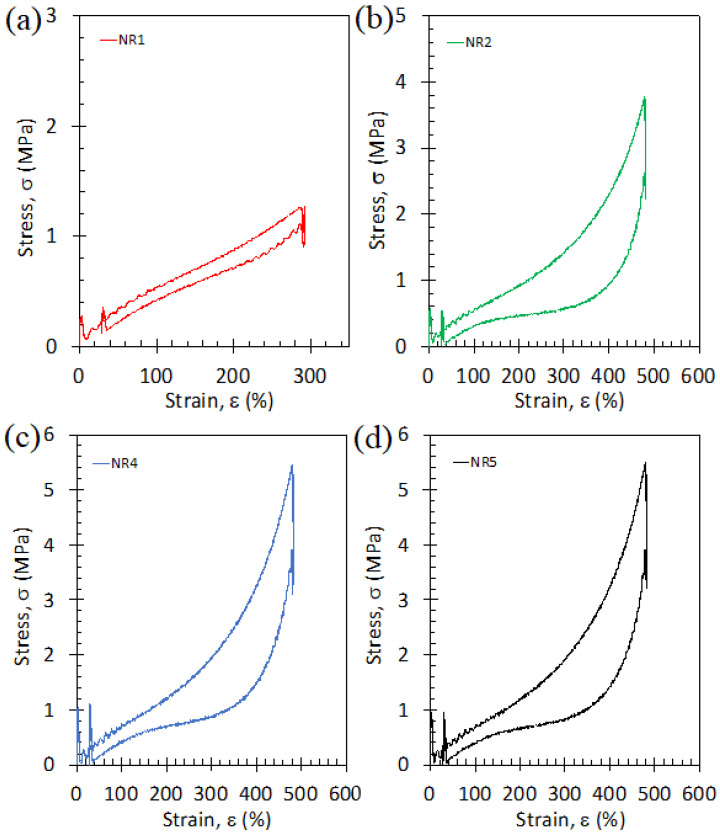
Stress–strain cycles performed at room temperature and at the strain rate of 3.33 s^−1^ for the specimens of different thicknesses, NR1 (**a**), NR2 (**b**), NR3 (**c**) and NR4 (**d**) as indicated in Table 1. Loading and unloading phases are separated by relaxation steps of 60 s.

Cyclic tensile curves are shown in Figure 7. For all materials, the unloading curve is found to be lower than the loading one revealing a mechanical hysteresis that is mostly induced by a “superstraining” effect (analogous to the supercooling effect seen in thermal crystallization). This phenomenon is because the strain at crystallization onset is always found to be higher than the strain required to complete melting. This is due to the difference in nature between crystallization which requires the nucleation of a new phase (the rubber materials are 100% amorphous in the undeformed state at room temperature) and hence a supplementary deformation to jump the nucleation barrier energy, while the melting occurs in a semi-crystalline rubber where the amorphous/crystalline interface is already present. 

**Figure 8:**
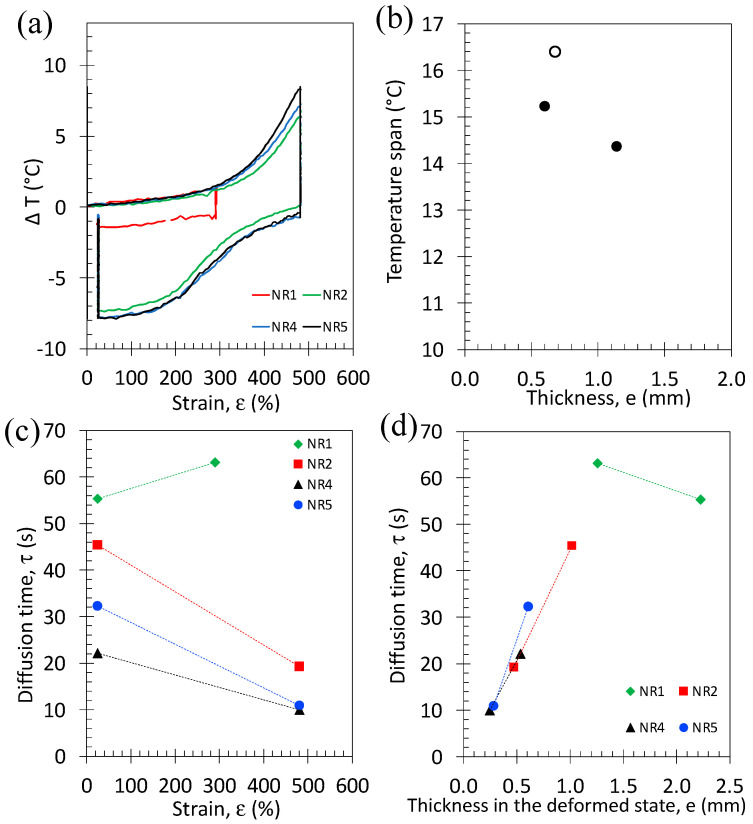
(**a**) Temperature change during high strain rate cycles, (**b**) the temperature span (maximum temperature difference between the maximum reached by the material after the loading and the minimum after the unloading, (**c**) thermal diffusion time versus strain, and (**d**) thermal diffusion time versus specimen thickness in undeformed and deformed states.

From the mechanical hysteresis, we may expect a dissymmetry in terms of deformation level in the crystallization and melting processes. This is traduced by a hysteretic behavior between the temperature rise during the loading, mostly due to crystallization, and the temperature drop during unloading (Figure 8a). The temperature span, DT (maximum difference in temperature between loading and unloading phases), characterizes the overall heating/cooling capacity of the material during a cycle. The latter is found to increase while decreasing the specimen thickness (Figure 8b), consistent with a higher reinforcement effect and, by inference crystallization ability due to a higher crosslink density, as seen in Section 3.1. The thermal diffusion time, τ, characterizes the time required to reach the equilibrium temperature after rapid loading or unloading. It is calculated via the equation shown in reference [33]. For all cycles, the thermal diffusion time is found to decrease with applied strain (Figure 8c), except for the material that has undergone small cycles (NR1). Such reduction of thermal diffusion time with strain is due to the decrease in the specimen thickness with the applied deformation. Assuming a conservation of the specimen volume during its loading along the longitudinal axis of the specimen (tensile axis), a reduction of the transversal section (thickness × width) by compression is observed. Assuming transversal isotropy (compression of the specimen thickness and width are similar), the thickness of the specimen in the deformed state can be calculated (the details of the calculation can be found in the reference [33]). The thermal diffusion time is found to increase linearly with the specimen thickness (Figure 8d). Only one data point does not obey this trend, which is the thermal diffusion time calculated for the small cycle, associated with a weak temperature change (DT = 2 °C) that is thought to be at the origin of such inconsistency.

**Figure 9:**
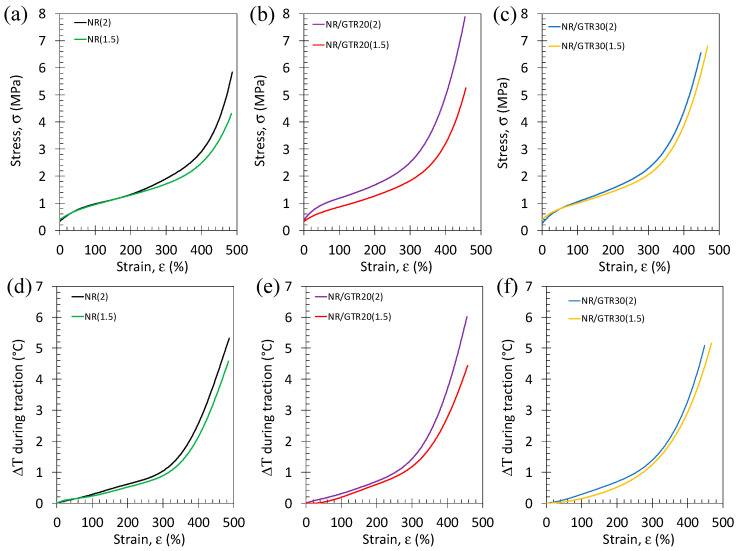
Stress–strain curves performed at room temperature and at the strain rate of 3.33 s^−1^ for the NR (**a**), NR/GTR20 (**b**) and NR/GTR30 (**c**) for two contents of the crosslinking agent, dicumyl peroxide (DCP): 1.5 wt.% and 2 wt.% of NR. Corresponding temperature variation versus strain measured during the same tests for the NR (**d**), NR/GTR20 (**e**), and NR/GTR30 (**f**).

**Figure 10:**
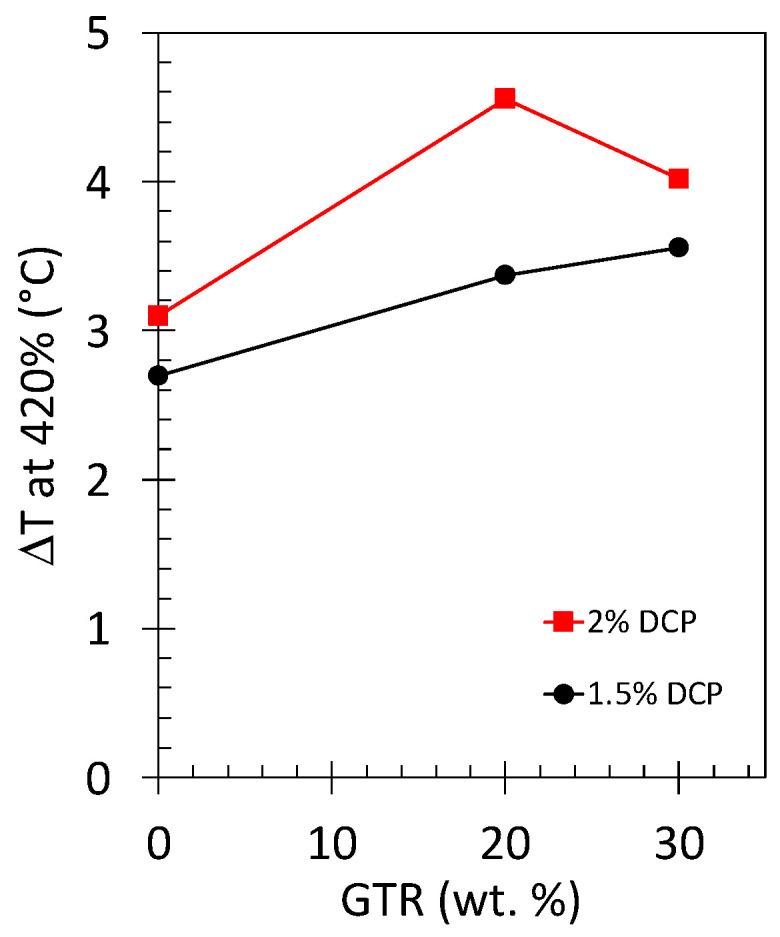
Temperature changes measured during loading at 420% of deformation for the NR, NR/GTR20, and NR/GTR30 for two contents of the crosslinking agent, dicumyl peroxide (DCP): 1.5 wt.% (filled circles) and 2 wt.% of NR (filled squares).

**Figure 11:**
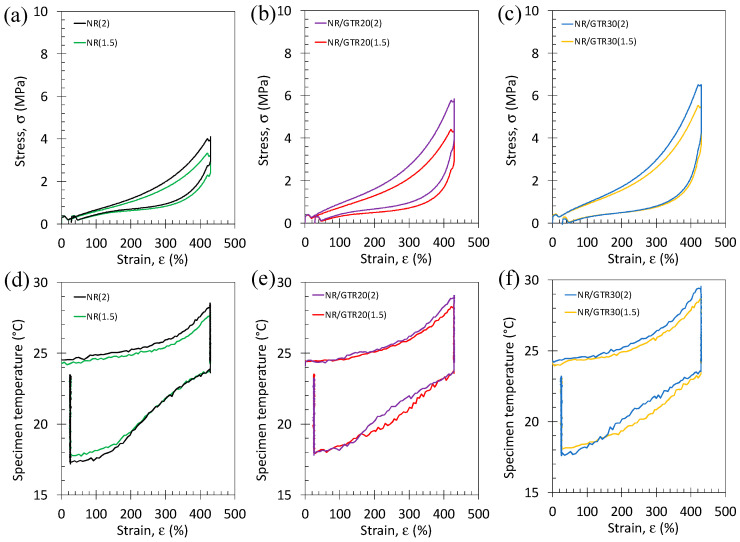
Stress–strain cycles performed at room temperature and at the strain rate of 3.33 s^−1^ for the NR (**a**), NR/GTR20 (**b**), and NR/GTR30 (**c**) for two contents of the crosslinking agent, dicumyl peroxide (DCP): 1.5 wt.% and 2 wt.% of NR. Corresponding temperature variation versus strain measured during the same tests for the NR (**d**), NR/GTR20 (**e**) and NR/GTR30 (**f**).

**Figure 12:**
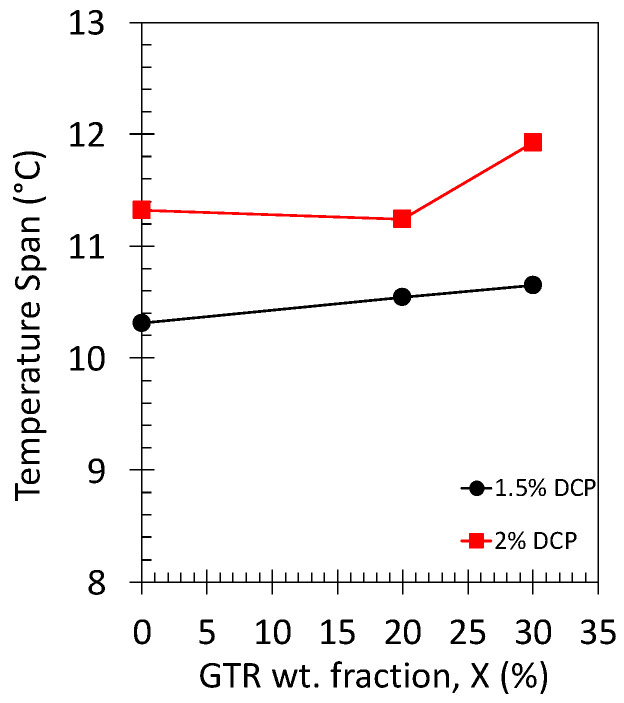
Temperature span (maximum difference in temperature measured during the loading and unloading) for the NR, NR/GTR20, and NR/GTR30 for two contents of the crosslinking agent, dicumyl peroxide (DCP): 1.5 wt.% (filled circles) and 2 wt.% of NR (filled squares).

The effect of the amount of vulcanizing agent and of GTR on the eC behavior in NR and NR/GTR blends were investigated under single loading (Figure 9 and Figure 10) and cyclic loading (Figure 11 and Figure 12). As for the slow strain rates, the increase in the amount of vulcanizing agent tends to increase the mechanical reinforcement in NR, but this effect is not pronounced for the NR/GTR30 blend (Figure 9a–c). Such changes in the reinforcement occur at a strain range above 300%, while the low strain range (below 300%) are not deeply affected. As this large strain reinforcement had been shown to be related to the occurrence of strain-induced crystallization (SIC), we may assume here that the main effect of the vulcanizing agent is the supplementary nucleating effect on SIC. As what was observed on the stress–strain curves, the temperature change at the specimen surface is not widely modified by the presence of the DCP at moderate strain (below 300%), but at large strain (above 300%), the rise in temperature more significantly varies (Figure 9d–f). The increase in DCP content is associated with a more rapid rise in temperature. Once again, this effect almost vanishes for the NR/GTR blends using 30 wt.% of GTR.

To better quantify the effect of the DCP content on the heating property of the NR and NR/GTR materials, the temperature change measured at a deformation of 420%, DT_420%_, has been measured. DT_420%_ is found to increase while increasing the quantity of vulcanizing agent (i.e., increasing the crosslink density), showing an increasing effect of the DCP from NR to NR/GTR20 blends (Figure 10). At higher content (30 wt.%), the DCP effect is less pronounced. As was stated above, this is supposed to be due to an underestimate of the DCP content required to fully vulcanize not only the NR matrix and at the GTR surface but also in the GTR volume.

Fast stress–strain cycles were performed on NR/GTR blends with the two amounts of DCP. Tensile curves show a hysteretic behavior induced by the dissymmetry between the crystallization and melting occurring during loading and unloading, respectively (Figure 11a–c). A significant increase in the area under the tensile curve with the addition of DCP particles can be noted. This increase in hysteresis may be due to the higher ability of highly crosslinked blends (2 wt.% DCP) to generate the strain-induced crystal phase during loading by increasing the number of nucleating points. Heating is observed during loading and cooling during unloading, showing a hysteresis behavior. The loading phase shows an increase in heating during loading while increasing the DCP content, while the unloading phase does not seem to show a clear trend (Figure 11d–f).

To better characterize the heating/cooling ability in the NR and NR/GTR blends, the temperature span, DT, was determined (Figure 12). DT is always found to be higher while using the highest DCP content, independently of the GR content. This result is mostly due to the highest crosslinking density in the materials containing 2 wt.% DCP that favors the crystallization process (higher facility to align the rubber chains along the stretching axis as the crosslinks act as pivots). The NR/GTR30 composite with the highest DCP appears to be the most efficient in terms of heating/cooling, with a maximum temperature span of more than 12 °C. 

Some questions remain, however, regarding both heating and cooling effects during the application of a series of cycles that would be required to provide its suitability in a heating/cooling device. Dissipative mechanisms, such as viscoelasticity and strain-induced crystallization/melting, may vary under multiple cycles and, hence, alter the heating/cooling ability.

**Figure 13:**
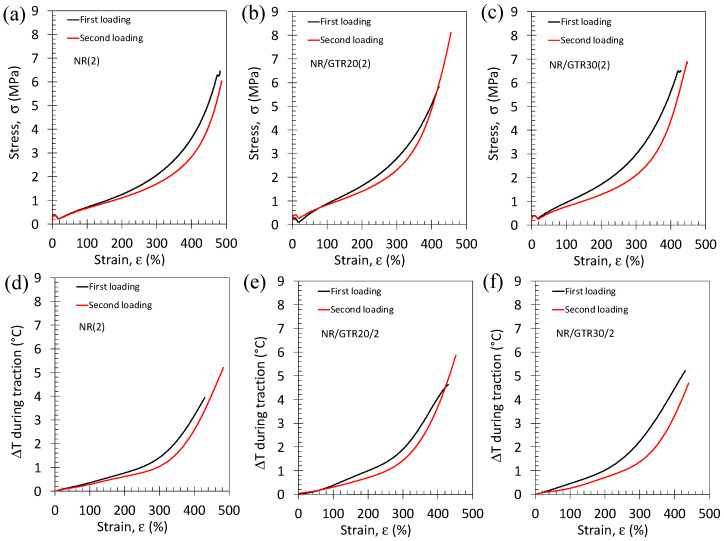
Loading test performed at room temperature and at the strain rate of 3.33 s^−1^ for the NR (**a**), NR/GTR20 (**b**) and NR/GTR30 (**c**) vulcanized with 1.5 wt.% of the NR phase. The black and red curves correspond to the first and second loading, respectively, performed on the same specimen. Corresponding temperature variation versus strain measured during the same tests for the NR (**d**), NR/GTR20 (**e**), and NR/GTR30 (**f**).

The mechanical and elastocaloric behavior of the first and second loading were compared for NR and NR/GTR with the two DCP contents (Figure 13). For each material, the first and second loading was performed on the same specimen with a relaxation time of at least 2 h between the two tests. For all materials, the second loading is found to be lower than the first one. This effect seems to be weekly dependent on the quantity of GTR or DCP.

**Figure 14:**
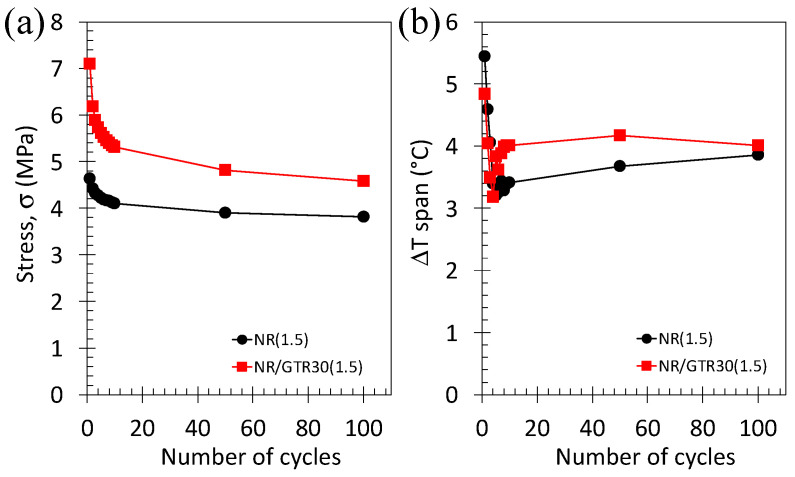
Maximum stress (**a**) and temperature span (**b**) during a cyclic test performed on NR (filled circle) and NR/GTR 30 (filled squares) containing 1.5 wt.% DCP a constant frequency of 0.3 Hz with an amplitude of strain of 200%, corresponding to a nominal strain rate of 120% s^−1^. Prior to the cyclic deformation, the rubber specimen is pre-deformed up to 400%.

While the materials with the highest crosslink density show the best elastocaloric performance under a single cycle, preliminary tests (unshown) indicate that the materials with the lowest DCP content (lowest density) can undergo the highest number of cycles. In the following, the application of multiple cycles will be applied to such materials. A series of 100 cycles has been performed on the reference vulcanized NR and on the NR/GTR30 wt.%. The materials have been first pre-stretched up to 200% of deformation, a deformation level sufficiently low to prevent SIC from occurring at that stage. Then, a cyclic test is performed between 200% and 400% to generate crystallization and melting during loading/unloading and, by inference, a heating/cooling effect due to their exothermic/endothermic nature. The maximum stress reached during each cycle and the temperature span (difference between the maximum and minimum temperature reached during a dynamic cycle) are presented (Figure 14). The stress is found to show a drastic drop within the first 10 cycles and then progressively stabilizes. This stress accommodation mechanism is more pronounced in the NR/GTR blends. While of different nature, such a mechanism appears in filled rubber and is denominated as the Mullins effect [49], that likely related to the chains slippage at filler surface and/or chains rupture between fillers aggregates. A similar mechanism has been evidenced on the stress and temperature changes in vulcanized NR [50]. In our materials, this effect may be induced by viscoelastic effect (e.g., disentanglement of rubber chains in the rubber matrix by accumulating mechanical cycles). Interestingly, the temperature change is found to be slightly higher in the case of NR/GTR blends as compared to the NR, suggesting these NR/GTR blends are potential candidates as performant eC materials. Nonetheless, the fatigue behavior of such materials should be characterized under more drastic conditions of solicitation (application of millions of cycles) to make them useful in heating/cooling prototypes. 

## 4. Conclusions

The elastocaloric (eC) effect in natural rubber (NR)/waste rubber blends was investigated under single and cyclic loading. The rubber specimen geometry (thickness), the quantity of vulcanizing agent (DCP), or the quantity of waste (ground tire rubber, GTR) were chosen to optimize the eC effect. Various testing conditions had been investigated, from laboratory testing conditions (slow strain rate single loading up to failure) to more realistic ones in terms of application in a heating/cooling device, i.e., continuous cycles at a high strain rate. 

The reduction of the specimen thickness (from 2.5 to 0.6 mm) was found to be associated with an increase in both the average value and homogeneity of the rubber chain’s crosslink density. This resulted in an increase in the large strain reinforcement, likely ascribed to an improvement of the strain-induced crystallization (SIC) ability. This resulted in an improvement of the associated eC effect under cyclic behavior at high strain rates, with a maximum temperature span of 16.5 °C for the thinnest material. The reduction of the rubber specimen thickness also showed the advantage of drastically decreasing the time of return to equilibrium temperature from 65 to 10 s during the relaxation phases of the cyclic tests. The increase in the amount of crosslinking agent in the NR and NR/GTR blends was also found to increase the large strain reinforcement as well as the eC effect under single and cyclic loadings at high strain rates, with a maximum temperature span of 12.1 °C in the NR/GTR30 containing 2 wt.% of DCP against 10.6 °C in the NR/GTR30 containing only 1.5 wt.% of DCP. 

Further improvement of the temperature span under continuous cyclic loading will be targeted for the application of such materials in heating/cooling prototypes. In future studies, the incorporation of thermally conductive fillers may be carried out to improve the heat transfer from the material to the room, and the rheology and mechanical properties of the resulting blends characterized for an optimization of the fillers spatial distribution to reach a percolating network.

## Figures and Tables

**Table 1:** Materials codes. * The NR_5_ material has been cured like other materials but with an additional cooling phase of −50 °C/minute to extend the vulcanization process. ** The DCP was introduced as a weight percent of the NR phase. GTR fraction expressed in 20 and 30 wt.% respectively corresponds to 25 and 47 phr (parts per hundred rubber), respectively. The quantity of DCP expressed in 1.5 and 2 wt.% of natural rubber matrix respectively correspond to 1.48 and 1.96 phr, respectively.

Material Code	Mat. Thickness (mm)	GTR (wt.%)	DCP (wt.%) **
NR_1_	2.57 (+/−0.04)	0	1.5
NR_2_	1.21 (+/−0.05)	0	1.5
NR_3_	1.03 (+/−0.03)	0	1.5
NR_4_	0.75 (+/−0.03)	0	1.5
NR_5_ *	0.75 (+/−0.03)	0	1.5
NR_(1.5)_	0.67 (+/−0.03)	0	1.5
NR_(2)_	0.69 (+/−0.03)	0	2
NR/GTR20_(1.5)_	0.62 (+/−0.04)	20	1.5
NR/GTR20_(2)_	0.64 (+/−0.02)	20	2
NR/GTR30_(1.5)_	0.62 (+/−0.02)	30	1.5
NR/GTR30_(2)_	0.63 (+/−0.02)	30	2

## Data Availability

Research data can be found at https://www.researchgate.net/profile/Nicolas-Candau (accessed on 22 May 2023).
